# Older adults' beliefs about physician-estimated life expectancy: a cross-sectional survey

**DOI:** 10.1186/1471-2296-7-9

**Published:** 2006-02-11

**Authors:** Christine E Kistler, Carmen L Lewis, Halle R Amick, Debra L Bynum, Louise C Walter, Lea C Watson

**Affiliations:** 1 Family Medicine, University of Michigan, 200 Arnet St., Suite 200, Ypsilanti, MI 48198-HCA, USA; 2 Medicine, University of North Carolina at Chapel Hill, Chapel Hill, USA; 3 Medicine Administration, University of North Carolina at Chapel Hill, Chapel Hill, USA; 4 Medicine, University of California, San Francisco, San Francisco, USA; 5 Psychiatry, University of North Carolina at Chapel Hill, Chapel Hill, USA

## Abstract

**Background:**

Estimates of life expectancy assist physicians and patients in medical decision-making. The time-delayed benefits for many medical treatments make an older adult's life expectancy estimate particularly important for physicians. The purpose of this study is to assess older adults' beliefs about physician-estimated life expectancy.

**Methods:**

We performed a mixed qualitative-quantitative cross-sectional study in which 116 healthy adults aged 70+ were recruited from two local retirement communities. We interviewed them regarding their beliefs about physician-estimated life expectancy in the context of a larger study on cancer screening beliefs. Semi-structured interviews of 80 minutes average duration were performed in private locations convenient to participants. Demographic characteristics as well as cancer screening beliefs and beliefs about life expectancy were measured. Two independent researchers reviewed the open-ended responses and recorded the most common themes. The research team resolved disagreements by consensus.

**Results:**

This article reports the life-expectancy results portion of the larger study. The study group (n = 116) was comprised of healthy, well-educated older adults, with almost a third over 85 years old, and none meeting criteria for dementia. Sixty-four percent (n = 73) felt that their physicians could not correctly estimate their life expectancy. Sixty-six percent (n = 75) wanted their physicians to talk with them about their life expectancy. The themes that emerged from our study indicate that discussions of life expectancy could help older adults plan for the future, maintain open communication with their physicians, and provide them knowledge about their medical conditions.

**Conclusion:**

The majority of the healthy older adults in this study were open to discussions about life expectancy in the context of discussing cancer screening tests, despite awareness that their physicians' estimates could be inaccurate. Since about a third of participants perceived these discussions as not useful or even harmful, physicians should first ascertain patients' preferences before discussing their life expectancies.

## Background

Estimates of life expectancy assist physicians and patients in medical decision-making [1]. The time-delayed benefits for many medical treatments, such as cancer screening, make an older adult's life expectancy estimate particularly important. To address this, the American Geriatrics Society and others recommend using life expectancy to guide medical decision-making in health screening [2-4].

Despite these recommendations, physician inaccuracy at predicting patients' life expectancies in a variety of illnesses has been detailed [5-9]. This inaccuracy has been attributed to factors such as physician uncertainty and personal optimism [10], both conscious and unconscious [11]. In one study, physicians overestimated survival by at least a factor of 5 in terminally ill patients [12]. When asked to respond to questions about life expectancy, a group of oncologists always responded with disclaimers about their predictions [13]. Therefore, while physicians are encouraged to use life expectancy estimates to guide decision making, they may feel unskilled, ill-equipped, and uncomfortable with the task [11,14-17].

Although physicians may feel uncomfortable making these estimates, many older adults may want estimates about their life expectancy from their physicians. The President's Commission for the Study of Ethical Problems in Medicine found that 75% of adults 65 and older preferred to be given a "realistic estimate" of their life expectancy [18]. In a recent survey of 205 terminally ill older adults, 55% reported wanting to discuss their life expectancy with their physicians [19]. Eighty-one percent of 2331 terminally ill older adults over the age of 70 reported that they wanted as much information as possible [20].

The literature in this area is sparse and primarily addresses older adults' desires for life expectancy estimates at the end of life, not in the context of screening decisions. Furthermore, it is unclear from previous studies whether older adults would want physicians to provide life expectancy estimates if they believe these estimates to be inaccurate. The purpose of this study is to assess healthy older adults' beliefs about physicians' ability to estimate their life expectancy and whether or not they desire to discuss their life expectancy with their physician given these beliefs.

## Methods

### Study design: Mixed qualitative-quantitative cross-sectional study

#### Participant recruitment

Participants were recruited from the independent living facilities of two local Continuing Care Retirement Communities (CCRCs) as these individuals were more likely to provide meaningful interviews and represented a sample population likely to have had discussions involving cancer screening and life expectancy. Unlike quantitative data collecting, a qualitative study does not rely on its power to ascertain sample size. Participants in our study continued to be enrolled until no new domains emerged and redundancy was reached. For our larger study, we had a target sample size of 100 to 150 participants which we projected would achieve statistical significance.

Flyers were placed in the clubhouses of the two local CCRCs where residents gather to collect their mail. Interested residents called a provided phone number of one of the researchers (CK) to volunteer. Age greater than 70 and residency in one of the independent living facilities were the only inclusion criteria. Exclusion criteria were any factors that would preclude a meaningful interview such as cognitive impairment, aphasia, profound hearing impairment, language barriers, or participation in pilot interviews. One interviewer performed all interviews in private locations convenient to the participant, usually the participant's home. All visually capable participants gave written informed consent. If the participant was visually impaired, the written consent was read aloud verbatim by the interviewer and the participant was consented in this manner.

The Institutional Review Boards at the University of North Carolina at Chapel Hill (UNC-CH) and the CCRCs approved the study protocols.

### Survey instrument

Questions and statements were developed by the authors and pre-tested in a population over 65 years of age from the UNC-CH Ambulatory Care Clinic. The questionnaire contained 22 items and probed a variety of issues regarding cancer screening beliefs and behavior, including life expectancy. The conceptual model used to develop the study instrument was a framework to assess cancer screening, from quantitative information such screening frequencies to the factors that influence cancer screening, including informed decision-making and perceptions of life expectancy. The more emotionally provocative questions and statements were addressed at the end of the questionnaire to avoid biasing further discussions using basic interviewing techniques [21]. The instrument was tested for understanding using cognitive interviewing skills [22] on 49 participants by two of the researchers (CK and HA). Participants were asked to explain each question or statement back to the researcher in their own words, to give their definitions for key terms, to help identify what particular parts of questions or statements gave them difficulty, and to offer advice on possible changes to the survey. Changes to the survey were made according to feedback after every 10–15 patients. Each of the iterations was then retested.

As a part of the comprehensive study, participants were asked to respond to an item on life expectancy that included two statements on a Likert response scale of "strongly disagree", "disagree", "agree", and "strongly agree". Each statement was followed by a probing question. The statements and questions were: 1) "I feel that my main doctor can correctly estimate how long I might live" followed by "Why do you feel that way?"; and 2) "I want my main doctor to talk to me about how long I might live", followed by "Why do you feel the way you do?".

Participant demographics were collected. Participants' physicians' demographics were collected to see if gender of physician and/or participant, length of physician-participant relationship, or physician specialty might influence participant beliefs. Current health status and global function were assessed with a comorbidity questionnaire, based on Katz [23], an Instrumental Activities of Daily Living (IADL) questionnaire [24], and a 6-item cognitive screen [25].

### Data collection and data analyses

Verbal responses were recorded on individual survey questionnaires by the interviewer. All open-ended responses were recorded verbatim in written notes by the investigator (CK). We used an integrated qualitative approach to data collection and a linked method approach to data analysis [26]. A "naïve" qualitative analysis of the data was performed. The analysis is "naïve" in the sense that we did not have a preconceived template as has been done in similar studies [27]. The transcripts will be read for themes and coded.

We used frequencies to describe the demographic characteristics and responses to our two Likert-scaled statements. The data was analyzed by an investigator (CK) and confirmed by another investigator independently (LW). Two investigators (CK and HA) independently coded the open-ended responses after the responses had been blinded. They identified common domains and presented them to the research team. Initial domains were identified during preliminary reading of the responses. The domains were refined and revised based on iterative readings by two investigators (CK and HA). Final domains were reviewed independently by the research group. Internal consistency for domains was assessed by independently rating the transcripts and comparing the coding. Coding discrepancies were discussed with the group and resolved by consensus.

Three domains emerged as most common in both participants' beliefs about the correctness of physician estimates and their desire to discuss these estimates.

## Results

Characteristics of the participants are shown in Table 1. The study group was comprised of healthy, well-educated older adults, with almost a third over 85 years old, and none meeting criteria for dementia. We also assessed several characteristics of the physicians identified by the participants as their primary physician. The 116 participants saw 34 different physicians. Of these physicians, 49% were women and 93% were generalists. Only 34% of participants had seen their physicians for more than 5 years.

**Table 1:** Participant characteristics (n = 116)

**Characteristic**	**N (%)**
Age: 70–84	80(69)
Age: > 85, maximum age: 96	36(31)
Female	78(67)
Married or living with partner	62(53)
Education: College graduate or higher	96(83)
Independent in IADLs*	110(95)
Health reported as excellent to very good	65(56)
Takes more than 4 prescriptions medicines per day	104(90)

*instrumental activities of daily living

### Beliefs in the accuracy of physicians' estimates of life expectancy

Sixty-four percent of participants (n = 73) either disagreed (49%) or strongly disagreed (15%) with the statement "I feel that my main doctor can correctly estimate how long I might live". Thirty-six percent (n = 41) either agreed (32%) or strongly agree (4%) with the statement. When asked to explain why, three major domains emerged in comments made by those who both agreed and disagreed. All domains and comments are displayed in Table 2. These domains were: 1) personal experience; 2) physician knowledge; and 3) uncertainty. Comments made by those who disagreed pointed out the complexity and uncertainty of such predictions.

**Table 2:** Representative participant responses to the "I feel that my main doctor can correctly estimate how long I might live" Likert statement by domains

Participants' response	Domain	Example
Agree or strongly agree	1) personal experience	1) "Because she knows my overall physical condition and hopefully she is knowledgeable and aware of her limitations. Nobody knows how long anybody is going to live; nobody can play God, but they do know for certain conditions and to a certain degree."
	2) physician knowledge	2) "They know for the most part. Partly because when my husband died suddenly, and he had just had a physical, so I went to see my doctor because I didn't want to die suddenly too, and he said I'd live 20 more years, but there are some conditions they don't have tests for, and you have to forgive them that
	3) uncertainty	3) "It's a guesstimate, you know. I had a horrendous car accident in September, and it's a miracle I did not die. It can happen fast and have nothing to do with disease."
Disagree or strongly disagree	1) personal experience	1) "They don't know. My sister defied the life-expectancy predictions. She had metastatic breast disease and was in a coma; now she's doing okay."
	2) physician knowledge	2) "I don't think medicine is that well-known. It's terribly complex."
	3) uncertainty	3) "I could get killed by an automobile tomorrow, or by having something fall on me."

In the 36% of participants who agreed that their doctor could estimate how long they might live, most acknowledged the limitations and uncertainty of making such predictions. No major separate domains emerged that differed between those who disagreed or agreed. Participants often cited more than one domain in their responses. Two participants refused to respond to the first Likert-scaled statement and were removed from only this particular statement but included in all other analyses.

### Preferences for discussions about physicians' estimates of life expectancy

**Figure 1:**
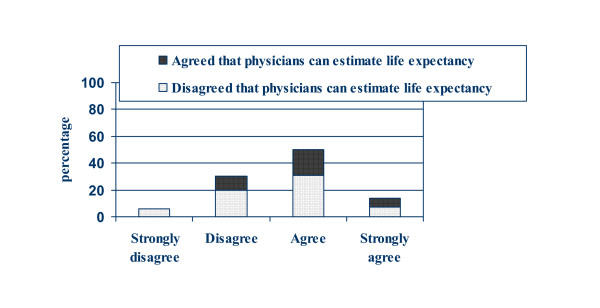
Participant responses to: "I want my main doctor to talk to me about how long I might live". (n = 114), stratified by participants' responses to "I feel that my main doctor can correctly estimate how long I might live."

Sixty-six percent of participants (n = 75) either agreed (52%) or strongly agreed (14%) with the statement "I want my main doctor to talk to me about how long I might live". Thirty-four percent (n = 41) either disagreed (29%) or strongly disagreed (5%). Figure 1 displays these results stratified by participants' response to the first Likert statement about their beliefs in the accuracy of physician-estimated life expectancy. Of note, the two non-responders to the first statement are not included in this percentage.

While a majority of participants wish to discuss life expectancy, not all of them believe their physicians' estimates, as shown by figure 1. Forty-three participants who want to discuss life-expectancy do not believe their physicians can correctly estimate their life expectancy.

When asked to explain why, three major domains emerged regarding participants' preferences in being told their life expectancy: 1) ability to plan; 2) communication preferences; and 3) knowledge preferences (Table 3). Comments made by those who wanted to have discussions with their physicians emphasize the positive effect of such information. Comments made by participants who did not want to have discussions demonstrate the potential negative effects of such information.

**Table 3:** Representative participant responses to the "I want my main doctor to talk to me about how long I might live" Likert statement by domains

Participants' response	Domain	Example
Agree or strongly agree	1) ability to plan	1) "If it's positive, if they think I'm going to live a long while, I could plan accordingly. It would help you get your house in order, so to speak, if it's negative, if I only have a little time left to live."
	2) communication preferences	2) "Because I have a right to know what he's thinking about, I wouldn't go to a doctor who wouldn't talk to me or communicate freely."
	3) knowledge preferences	3) "I want to know as much as I can about myself. I'm not afraid to be told I may die or when."
Disagree or strongly disagree	1) ability to plan	1) "It might discombobulate me and interfere with my will to live."
	2) communication preferences	2) "What for? What will be accomplished by that conversation, as long as I'm healthy? If I were seriously ill, I'd want to make provisions while I could, for my family."
	3) knowledge preferences	3) "It would color all of your decisions and I don't think you should be making them on a guesstimate!"

## Discussion

In this select population, we found that the majority of older adults did not believe their physicians could correctly estimate their life expectancy, but still wanted their physician to discuss their life expectancy with them. This study adds to the existing literature in two ways. First, although previous studies have documented that the majority of older adults desire to discuss life expectancy [18,20,28,29], they have not asked whether older adults believe these estimates to be accurate. Second, these studies were primarily conducted in the terminally ill. Our study, by contrast, assessed the perspectives of healthy older adults in the context of cancer screening decisions.

As cancer screening is one of the most emotionally charged topics in health care screening, our results may underestimate the number of patients willing to discuss life expectancy. More participants may have been willing to discuss life expectancy if it were in a less troublesome context, such as lipid or blood pressure screening. Participants' desire to discuss life expectancy even in relation to such a possibly worrisome subject shows how older adults find life expectancy discussions to be an acceptable part of the physician-patient relationship.

Our results also support the notion that some patients may benefit from discussions of life expectancy for reasons beyond medical decision-making. The themes that emerged from our conversations indicate that discussions of life expectancy could help older adults plan for the future, maintain open communication with their physicians, and provide them knowledge about their medical conditions.

On the other hand, a minority of participants had concerns about having these discussions. Among these was a group who would not believe the accuracy of physician estimates, and therefore would not find the information useful. Others voiced concerns about the effects on their emotional state or their will to live. For these individuals, these concerns could outweigh the potential benefits of having these discussions with their physicians. From these findings, physician reluctance to discuss life expectancy because it may harm the patient's outlook [14-16,19] appears to be warranted for some patients and points out the need for physicians to determine patient preference before discussing the issue.

This study was limited by the small number and select nature of participants. Therefore, these results should be considered preliminary and need to be replicated in a larger, more generalizable sample. Despite this limitation, the results are an important step in understanding the complexity of discussing life expectancy with older adults. Future studies are needed to explore these issues in older adults of differing social and economic backgrounds.

## Conclusion

This study expands previous findings by suggesting that healthy older adults may be open to life expectancy discussions and may benefit from them despite awareness that their physicians' estimates could be inaccurate. As approximately one third of patients perceived that these discussions could be harmful, physicians should first ascertain patient preferences before discussing their life expectancies.

## Abbreviations

CCRC: Continuing Care Retirement Community

UNC-CH: University of North Carolina at Chapel Hill

IADL: Instrumental Activities of Daily Living

## Competing interests

The author(s) declare that they have no competing interests.

## Authors' contributions

CLL conceived of the study. CEK, CLL, HRA, DLB, and LCWatson participated in the design of the survey. CEK and HRA tested the survey instrument and CEK carried out the interviews and drafted the manuscript. CEK, HRA, CLL, and LCWatson all contributed to the statistical analyses in the work. CEK, CLL, HRA, LCWatson, and LCWalters participated in the critical revisions of the manuscript. All authors read and approved the final manuscript.

## Pre-publication history

The pre-publication history for this paper can be accessed here:


